# Endoscopic diagnosis of chronic diarrhea

**DOI:** 10.1002/deo2.53

**Published:** 2021-09-28

**Authors:** Yoshikazu Kinoshita, Ryusuke Ariyoshi, Seiji Fujigaki, Katsuhide Tanaka, Teruhisa Morikawa, Tsuyoshi Sanuki

**Affiliations:** ^1^ Department of Medicine Hyogo Brain and Heart Center Hyogo Japan; ^2^ Department of Gastroenterology Steel Memorial Hirohata Hospital Hyogo Japan

**Keywords:** amyloidosis, bile acid diarrhea, celiac disease, collagenous colitis, eosinophilic gastroenteritis

## Abstract

The prevalence of chronic diarrhea in the general population is reported to be 4%–5%. Since various pathological conditions cause diarrheal symptoms, etiological diagnosis of chronic diarrhea is difficult in many cases. Medical history taking, physical examinations, and laboratory testing are not adequately sensitive or specific, thus a colonoscopic investigation is frequently employed for etiological evaluation. However, for cases with non‐bloody chronic diarrhea, the diagnostic yield of a colonoscopy procedure is reported to be not high enough. Furthermore, endoscopically identifiable findings are not adequately specific for the diagnosis of diarrheal disease, except for inflammatory bowel disease, while microscopic colitis, amyloidosis, eosinophilic gastroenteritis, celiac disease, and bile acid diarrhea are difficult to definitively diagnose using endoscopic findings. Thus, a histopathological examination of biopsy samples obtained with endoscopy is critically important. Endoscopists should consider obtaining biopsy samples from even normal‐appearing gastrointestinal mucosa for chronic diarrhea diagnosis.

## INTRODUCTION

Diarrhea is defined as a disorder in which water content in the stool is increased, resulting in stool softening and frequent bowel movements. It frequently develops in association with a pathological condition and in the majority of those cases is acute, with a duration of less than 4 weeks.^1^ Diagnosis of acute diarrheal disease is not difficult, since the possible pathogeneses are mainly limited to infectious, toxic, and food‐related etiologies. On the other hand, the pathogenesis of chronic diarrhea lasting over 4 weeks can be diverse and complicated, making etiological diagnosis difficult in some cases.^2^

Upper and lower gastrointestinal endoscopic investigations are frequently employed for differential diagnosis of chronic diarrhea. Endoscopy is useful to reveal organic gastrointestinal diseases, including neoplastic and inflammatory bowel diseases (IBDs). Furthermore, guidelines for the investigation of chronic diarrhea published by the British Society of Gastroenterology recommend a colonoscopy procedure to exclude colon cancer and microscopic colitis.^3^ Endoscopy findings are not adequate for identifying functional disease that causes diarrhea, thus an endoscopic study performed for the diarrheal disease should be done with special caution so as to increase its diagnostic value.

In this review article, appropriate endoscopic methods for the diagnosis of cases with chronic diarrhea are discussed.

## EPIDEMIOLOGY OF CHRONIC DIARRHEA

The prevalence of chronic diarrhea is reported to range from 4%–5% in the general population and is approximately half of chronic constipation.^3^, ^4^ Different from chronic constipation, which is more prevalent in elderly individuals, the prevalence of chronic diarrhea is higher in younger ages. The most frequently found organic disease identified in endoscopic studies is reported to be IBD, followed by microscopic colitis. IBD as well is more prevalent in young adults, while microscopic colitis, including collagenous colitis and lymphocytic colitis, is most frequently found in middle‐aged individuals and elderly females.^5^, ^6^

A list of diseases associated with chronic diarrhea is shown in Table 1. Causative pathologic conditions can be divided into chronic infectious diseases, IBDs, and related conditions, neoplastic diseases including hormone‐secreting tumors, malabsorption syndromes, drug‐related diarrhea, endocrine and metabolic diseases including hyperthyroidism, and functional gastrointestinal diseases including irritable bowel syndrome. Several of these are rarely encountered and diagnosis is not easily determined, even after performing a complete medical history and physical examination, as well as laboratory tests of blood, urine, and stool samples, and a colonoscopy procedure.^3^, ^7^

**TABLE 1:** Etiologies for chronic diarrhea

**Chronic infectious diseases**
Bacterial and fungal infection (intestinal tuberculosis, Whipple's disease, Yersinia infection, histoplasma infection, cap polyposis, small intestinal bacterial overgrowth, and so forth), parasitic infection (amebic colitis, Giardia lamblia, *Strongyloides stercoralis* infection, cyclospora infection, and so forth), viral infection (cytomegalovirus infection, AIDS enteritis, and so forth).
**Inflammatory bowel diseases**
Crohn's disease, ulcerative colitis, eosinophilic gastroenteritis, Behçet's disease, celiac disease, diverticular‐associated colitis, radiation colitis, and so forth.
**Neoplasms**
colon cancer, gastrointestinal malignant lymphoma, VIP oma, somatostatinoma, gastrinoma, and so forth.
**Malabsorption syndrome**
chronic pancreatitis, lactose intolerance, dumping syndrome, blind‐loop syndrome, pancreas cancer, and so forth.
**Irritable bowel syndrome and functional diarrhea**
**Drug‐related diarrhea**
Drug‐induced collagenous colitis (non‐steroidal anti‐inflammatory drugs, proton pump inhibitors, angiotensin receptor antagonists), ursodeoxycholic acid, lactulose, antibiotics, DPP4 inhibitors, immune checkpoint inhibitors, and so forth.
**Others**
Hyperthyroidism, diabetes mellitus, hypoparathyroidism, adrenal insufficiency, amyloidosis, alcohol consumption, bile acid diarrhea, post‐cholecystectomy, hypogammaglobulinemia, intestinal lymphangiectasis, short bowel syndrome, mesenteric ischemia, overflow diarrhea, microscopic colitis, and so forth.

## DIAGNOSIS OF CHRONIC DIARRHEA: MEDICAL HISTORY TAKING

**FIGURE 1:**
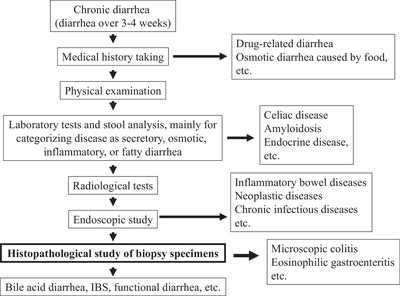
Flow of diagnosis of chronic diarrhea. IBS, irritable bowel syndrome

A complete medical history of an affected patient is important and can provide clues for the diagnosis of chronic diarrhea, the same as with other diseases (Figure 1). Information regarding chronic usage of drugs is critical for cases with drug‐related chronic diarrhea. Laxatives such as lactulose are rarely prescribed for the prevention of hepatic coma and can cause diarrhea, while bile acid administration for biliary diseases such as primary biliary cholangitis may also be causative. Additionally, nonsteroidal anti‐inflammatory drugs, selective serotonin reuptake inhibitors, and proton pump inhibitors are reported to be associated with microscopic colitis.^8^, ^9^ Dietary habits also should be checked, as excessive intake of caffeine or fermentable oligo‐, di‐, monosaccharides, and polyols including artificial sweeteners can be related to diarrhea.^3^

Past history of disease is also important. Radiation therapy to the lower abdomen, such as for uterine cervical cancer, as well as surgical excision of the lower ileum and a cholecystectomy, are known risk factors related to bile acid diarrhea, which is caused by decreased absorption of bile acids from the terminal ileum and increased hepatic production of bile acids.^4^ Habitual drinking of alcoholic beverages and possible chronic pancreatitis are risk factors for malabsorption‐related diarrhea.^10^ Furthermore, a history of chronic inflammatory diseases, such as tuberculosis or rheumatoid arthritis, suggests the possible presence of gastrointestinal amyloidosis and related diarrhea.^11^ Also, one‐third of patients with amyloid A amyloidosis are reported to have intractable diarrhea.^12^

## DIAGNOSIS OF CHRONIC DIARRHEA: PHYSICAL EXAMINATION

The role of a physical examination for the investigation of chronic diarrhea is limited.^2^, ^3^ Elevated body temperature suggests the presence of an inflammatory disease that can cause diarrhea, and patients with a chronic infectious disease or IBD are expected to have a fever. Those affected by hyperthyroidism may also show a low‐grade fever in addition to increased pulse rate, perspiration, and thyroid enlargement.

Scars on the abdominal wall indicating previous surgical treatment may suggest blind‐loop syndrome, dumping syndrome, bile acid diarrhea, or malabsorption syndrome and even small scars caused by laparoscopic surgery should be carefully checked. Patients with IBD, Behçet's disease, or celiac disease may be affected by skin eruption or arthritis, while skin melanin pigmentation can be related to adrenal insufficiency.

A careful abdominal physical examination may show tenderness and/or abnormal peristaltic sounds in inflammatory disease cases, and meteorism may be found in cases with malabsorption or impaired gut transit. A rectal digital examination can detect rectal tumors or fecal mass, and may suggest overflow diarrhea.

## DIAGNOSIS OF CHRONIC DIARRHEA: LABORATORY TESTS

Complete blood cell counts, blood biochemical testing, examinations of inflammatory markers including C‐reactive protein, thyroid functions, vitamin B12, and folate, and laboratory tests of stool samples are important first examinations that should be done. Different from western countries, a serologic test for celiac disease may not be mandatory as part of the initial diagnostic step in Japanese patients.^13^, ^14^ Anemia, especially iron‐deficiency anemia, suggests continuous hemorrhaging from the gastrointestinal tract, and suggests the presence of inflammatory or neoplastic gut disease. Patients with celiac disease may have iron‐deficiency anemia as well. Macrocytic anemia can indicate vitamin B12 deficiency caused by blind‐loop syndrome, short bowel syndrome, and/or small intestinal bacterial overgrowth. Elevated leukocyte number might also suggest inflammatory disease, and elevated peripheral eosinophile number can indicate eosinophilic gastroenteritis or parasitic infection such as strongyloidiasis.

Patients with diarrhea may also show dehydration and abnormal electrolyte concentration. VIPoma, gastrinoma, and adrenal insufficiency are accompanied individually by hypopotassemia, hypochlorhydria, and hyperpotassemia. Hypercalcemia accompanied by hypophosphatemia and hyperchlorhydria is an important sign of primary hyperparathyroidism. Patients with diabetes mellitus show hyperglycemia, while adrenal insufficiency may be accompanied by hypoglycemia. Decreased albumin and total protein concentrations can be found in cases of malabsorption syndrome including celiac disease and pancreatic insufficiency. Furthermore, hypoproteinemia may also occur in cases with protein‐losing gastroenteropathy, which can be found in those with IBD including eosinophilic gastroenteritis.

Fecal tests, such as gross inspection, microscopic observations, measurement of electrolytes, fecal pH measurement, fat content measurement, and stool occult blood testing, are useful to classify the case as secretory, osmotic, inflammatory, or fatty diarrhea (Table 2).^2^ Measurement of fecal electrolytes and calculation of osmotic gap can differentiate between secretory and osmotic diarrhea. The presence of fresh blood in the stool suggests hemorrhaging from the distal gut caused by inflammatory or neoplastic disease, while occult blood in the stool similarly suggests an inflammatory or neoplastic organic gastrointestinal disease. Fecal calprotectin measurement may be effective to detect inflammatory gut disease, while the presence of mucous can also indicate inflammatory disease. Findings of increased fat, and decreased fecal pH and glycogen concentration in the stool suggest malabsorption.^15^

**TABLE 2:** Categorization of chronic diarrhea

**Osmotic diarrhea**
Lactose intolerance, poorly absorbed foods, osmotic laxatives (lactulose, etc.), and so forth.
**Secretory diarrhea**
Microscopic colitis, bile acid diarrhea, post‐cholecystectomy, administration of bile acids, hyperthyroidism, diabetes mellitus, VIPoma, gastrinoma, somatostatinoma, adrenal insufficiency, irritable bowel syndrome, functional diarrhea, and so forth.
**Inflammatory diarrhea**
amebic colitis, cytomegalovirus infection, giardia lamblia, tuberculosis, Yersinia infection, histoplasma infection, cap polyposis, *Strongyloides stercorlis* infection, cyclospora infection, Crohn's disease, ulcerative colitis, eosinophilic gastroenteritis, Behçet's disease, diverticular‐associated colitis, radiation colitis, colon cancer, gastrointestinal malignant lymphoma, immune checkpoint inhibitor‐related colitis, and so forth.
**Fatty diarrhea**
Small intestinal bacterial overgrowth, blind‐loop syndrome, Whipple's disease, celiac disease, chronic pancreatitis, pancreas cancer, mesenteric ischemia, short bowel syndrome, and so forth.

In addition to routine laboratory tests, measurements of hormones, bile acids, tumor markers, and various antibodies may also be necessary for the diagnosis of more rare etiologies of chronic diarrhea.

## DIAGNOSIS OF CHRONIC DIARRHEA: RADIOLOGICAL EXAMINATIONS

Radiological examinations including plain abdominal radiology, a barium meal study, and computed tomography can reveal information for the diagnosis of organic diarrheal disease, though the diagnostic yield of these radiological examinations has yet to be clearly shown. Recently, magnetic resonance enterography is increasingly used for small bowel imaging.^16^

## DIAGNOSIS OF CHRONIC DIARRHEA: ENDOSCOPIC EXAMINATIONS

An endoscopic examination is widely used to reveal an organic disease as an etiological pathologic condition related to chronic diarrhea. For detecting neoplastic and inflammatory diseases, endoscopy is a powerful tool. However, the diagnostic yield of a routine colonoscopy examination has been reported to be as low as 15%–30%, even when an appropriate histopathological examination is added with multiple biopsy specimens.^17^, ^18^ In colonoscopic studies performed for patients with non‐bloody chronic diarrhea, microscopic colitis is the most frequently found disease, followed by IBDs including Crohn's disease and ulcerative colitis.^19^ Endoscopic diagnosis of ulcerative colitis, Crohn's disease, Behçet's disease, and diverticular colitis is possible, based on their characteristic endoscopic abnormalities when the terminal ileum is observed by colonoscopy. However, isolated ileitis can be missed when the terminal ileum is not observed during a colonoscopic examination.

Video capsule endoscopy has been reported useful for the diagnosis of cases with chronic diarrhea. According to a study performed in Korea, the rate of diagnosis was improved to 34.1% of patients who underwent a video capsule endoscopy examination.^20^ Also, in a multicenter study conducted in Greece, the diagnostic yield of video capsule endoscopy in patients with diarrhea, abdominal pain, and positive inflammatory markers was reported to be 90.1%.^21^ Therefore, capsule endoscopy may be considered for evaluation of ileal lesions as part of the work‐up for patients affected by chronic diarrhea.

The addition of a histopathological examination for ulcerative colitis and Crohn's disease is useful for diagnosis, as well as severity grading. Biopsy specimen sampling from gastrointestinal lesions identified by endoscopy is nearly routine practice during an endoscopic examination. However, when no identifiable lesion is found, the endoscopist may choose to not take biopsy specimens from the apparently normal mucosal surface. Some microscopic colitis cases show various types of abnormal mucosal lesions, including longitudinal mucosal tears and scars. On the other hand, in the majority of cases, no endoscopically identifiable colonic mucosal lesions can be found.^19^, ^22^ In these patients, routine multiple biopsy sampling from different colonic segments including the terminal ileum without an apparent lesion is an important diagnostic process to be added. When multiple samples are obtained and properly processed, histopathological diagnosis is not difficult. Thus, biopsy sampling from endoscopically normal‐appearing mucosa is an important addition for an accurate diagnosis of patients with chronic diarrhea of unidentified etiology.^23^

### Endoscopic biopsy important for the diagnosis of chronic diarrhea

Obtaining multiple biopsy samples from gastrointestinal mucosa is important for the diagnosis of microscopic colitis, amyloidosis, eosinophilic gastroenteritis, and celiac disease since an endoscopic examination is not sensitive enough to detect characteristic findings associated with these diseases (Table 3).

**TABLE 3:** Etiologies of chronic diarrhea for which diagnosis endoscopic biopsy sampling from apparently normal gastrointestinal mucosa is necessary

Eosinophilic gastroenteritis
Microscopic colitis (collagenous colitis and lymphocytic colitis)
Amyloidosis
Celiac disease
Functional bowel disease (irritable bowel syndrome, functional diarrhea, and bile aid diarrhea)

#### Microscopic colitis

Microscopic colitis, consisting of collagenous colitis and lymphocytic colitis, is frequently encountered as a cause of chronic diarrhea, with prevalence higher in middle‐aged and elderly individuals. Although various medications including nonsteroidal anti‐inflammatory drugs, selective serotonin reuptake inhibitors, angiotensin receptor antagonists, and proton pump inhibitors are reported to be related to the occurrence of collagenous colitis, the pathogenesis of microscopic colitis has not been completely elucidated.

For diagnosis of microscopic colitis, multiple colonic mucosal biopsy samples are necessary.^19^, ^22^ Once a histopathological examination of those biopsy samples is done, a thick subepithelial collagen band or dense lymphocyte infiltration can be detected, and microscopic colitis diagnosis confirmation is not difficult.^24^

#### Gastrointestinal amyloidosis

Amyloidosis is caused by tissue deposition of insoluble fibrillar abnormal protein. Presently, over 50 types of proteins have been reported as causes of amyloidosis. Gastrointestinal deposition of amyloid protein causes gastrointestinal motor abnormality and impaired blood perfusion, and affected patients mainly complain of diarrhea.^25^ Malabsorption and protein‐losing gastroenteropathy may also be found. AL type amyloid is reported to accumulate in submucosal and muscle layers and form nodules, while AA type amyloid accumulates in mucosal and submucosal tissue around the vessels or in patches in the gastrointestinal tract.

Endoscopic observation can detect various abnormalities including nodules, mucosal thickening, villous atrophy, ulcers, and mucosal color changes, though such abnormal findings cannot be found in many cases.^26^ Endoscopic biopsy sampling with appropriate histopathological testing is important for accurate diagnosis.

#### Eosinophilic gastroenteritis

Eosinophilic gastroenteritis is part of a subgroup of chronic delayed‐type allergic diseases termed eosinophilic gastrointestinal diseases. Although all gastrointestinal segments can be involved, the most frequently involved segment is the small intestine.^27^ The pathogenesis of eosinophilic gastroenteritis has not been completely clarified, though Th2‐type allergic response to intra‐luminal allergens including foods and those in the gut microbiome is considered to be the main etiological factor.^28^ This disease has been found in all age groups and both genders with similar rates of prevalence reported to range from 2–8 per 100,000 in the general population in western countries. Prevalence may be higher in Japan, though an appropriate etiological study has not been presented.^29^

Complaints of new patients are based on the gastrointestinal segments involved. Those with gastric involvement frequently note epigastralgia and nausea/vomiting, while patients with intestinal lesions frequently complain of abdominal pain and diarrhea.^27^ Eosinophilic gastroenteritis is a disease associated with chronic and intermittent diarrhea. Peripheral blood testing is useful when there is clinical suspicion of its presence, as 80% of affected cases show peripheral eosinophilia.^27^ Fecal calprotectin and eosinophil‐derived neurotoxin may be useful for the diagnosis and activity grading of eosinophilic gastroenteritis, though the study is still in progress.

Abdominal computed tomography can often detect the presence of ascites and segmental wall thickening of the involved gut. While a radiological examination is useful for examination of the small intestine, the most frequently involved segment of the gastrointestinal tract, endoscopic observation can detect various types of abnormalities, including mucosal edema and redness, erosions, ulcers, polypoid lesions, nodules, and narrowing. Since these abnormalities are non‐specific findings, diagnosis of eosinophilic gastroenteritis based only on endoscopic abnormalities is difficult. In addition, over 60% of cases with eosinophilic gastroenteritis are reported as not having gastrointestinal lesions identifiable by endoscopy.^30^ Therefore, multiple biopsy sampling and careful examinations of eosinophil infiltration of appropriately processed specimens are critically important. Accurate diagnosis of eosinophilic gastroenteritis is done based on clinical symptoms as well as histopathological identification of increased eosinophil infiltration in the mucosal layer.^29^ In addition to that increased infiltration, other histopathological abnormalities, such as intraepithelial eosinophils and epithelial cell damage, are known to be important.

#### Celiac disease

Celiac disease is caused by immune‐mediated small intestinal mucosal damage. In affected patients, antibodies against gliadin and tissue transglutaminase are frequently noted, a useful finding for diagnosis. These autoantibodies may have an important role in the destruction of the small intestinal villous structure with resulting malabsorption of nutrients, which are absorbed from the jejunum and upper ileum. Although the prevalence of celiac disease is reported to be rapidly increasing in western countries, its prevalence in Japan is still not high, probably because of the low prevalence of HLA DQ2 and DQ8, and lower wheat consumption. We performed an investigation of the prevalence of celiac disease in cases with diarrhea‐predominant abdominal symptoms. Of 46 patients with abdominal symptoms, only one case with celiac disease was found, while among 2000 individuals in the general population who underwent an annual health check and reported no bothersome symptoms that decreased their quality of life, three suspected and one definite cases were found.^13^, ^14^ These findings suggested that the prevalence of celiac disease in Japan is still quite low, even among cases of chronic diarrhea.

Patients with celiac disease may complain of malnutrition, diarrhea, and/or abdominal discomfort in advanced stages. Laboratory tests are useful for diagnosis, with the presence of the anti‐tissue transglutaminase IgA antibody a sensitive and specific diagnostic marker of celiac disease. An endoscopic study, especially duodenal observation, is important for diagnosis since the upper small intestine is the most frequently and strongly damaged segment of the gut. Villous atrophy of the duodenal mucosa is the most frequently encountered endoscopic finding, however, identification of that only by endoscopic observation is not easy,^31^ thus a histopathological examination of biopsy specimens is important. In addition to villous atrophy, hyperplasia of pits and increased intraepithelial lymphocytes are important findings for confirming the diagnosis.

For patients with chronic diarrhea, an upper gastrointestinal endoscopic study and multiple biopsy sampling of duodenal mucosa followed by complete histopathological testing are necessary, even in Japan, when other causes are ruled out. However, it is important to point out that the prevalence of celiac disease in Japan is low, thus the diagnostic yield of that method is also low.

#### Bile acid diarrhea

Laboratory tests of peripheral blood and stool, as well as upper and lower gastrointestinal endoscopic studies with multiple biopsy sampling followed by an appropriate histopathological examination often do not suggest the presence of organic diseases in cases with chronic diarrhea. In these, a diagnosis of diarrhea‐type irritable bowel syndrome will be given when the patient complains of abdominal pain. Similarly, a diagnosis of functional diarrhea will be given when there is no complaint of abdominal pain. Recent studies suggest that at least one‐third of these cases are not diarrhea‐type irritable bowel syndrome nor functional diarrhea, but rather bile acid diarrhea.^32^

Bile acids are secreted from the liver to the gastrointestinal tract through the biliary system, with over 95% absorbed mainly from the terminal ileum and the remaining small amount going into the colon. When a larger amount of bile acids enters the colon, colonic peristaltic contraction and water secretion are likely to be stimulated, and diarrhea occurs. Since the amount of bile acids that enter the colon is under the control of their active absorption in the distal ileum, decreased ileal absorption is expected to cause diarrhea. When bile acid absorption is decreased, hepatic de novo production of bile acids is stimulated. Chronic diarrhea caused by decreased absorption of bile acids in the distal ileum and overproduction of hepatic bile acids with a resulting increase in colonic bile acids load leads to bile acid diarrhea.

Symptoms noted by patients with bile acid diarrhea are similar to those related to other types, thus diagnosis based on the symptom complex is difficult and diagnostic methods have been developed. Among available tests, a ^75^selenium homocholic acid taurine test for determining body retention of an orally administered radioisotope‐labeled bile acid is reported to be the most accurate, though not presently available in Japan. Unfortunately, other tests, including those used to measure plasma 7α‐hydroxy‐4‐cholesten‐3‐one, plasma fibroblast growth factor 19, and stool bile acid, lack sensitivity. Thus, empirical administration of bile acid sequestrants and assessment of diarrhea during that administration is widely employed. Approximately 90% of cases with bile acid diarrhea will respond to a bile acid sequestrant. Since many cases of bile acid diarrhea are reported to be erroneously diagnosed as diarrhea‐type irritable bowel syndrome or functional diarrhea, such empirical administration of bile acid sequestrants is recommended for cases that show resistance to standard anti‐diarrhea treatment.

## SUMMARY

Chronic diarrhea is a frequently encountered condition, though the etiology is not easily identified in some cases. Precise history taking followed by a physical examination has limited diagnostic value, and the diagnostic yield of laboratory blood and stool testing is not high enough. In such cases, endoscopy is the best diagnostic examination as the next step. Using colonoscopy, IBDs can be diagnosed by their characteristic endoscopic abnormalities. However, the diagnostic yield of colonoscopy is not adequate for microscopic colitis, amyloidosis, eosinophilic gastroenteritis, and celiac diseases, for which endoscopic biopsy specimens may be useful and histopathological examination results will add important information for confirming the diagnosis. Endoscopists should consider obtaining biopsy samples even from endoscopically normal‐appearing gastrointestinal mucosa for accurate diagnosis of chronic diarrhea.

## CONFLICT OF INTEREST

The authors declare that they have no conflict of interest.

## FUNDING INFORMATION

None.
